# Modelling carbon stocks and fluxes in the wood product sector: a comparative review

**DOI:** 10.1111/gcb.13235

**Published:** 2016-03-04

**Authors:** Pau Brunet‐Navarro, Hubert Jochheim, Bart Muys

**Affiliations:** ^1^ Leibniz Centre for Agricultural Landscape Research (ZALF)Institute of Landscape Systems AnalysisEberswalder Straße 8415374MünchebergGermany; ^2^ Division Forest, Nature and LandscapeUniversity of LeuvenCelestijnenlaan 200E‐2411BE‐3001LeuvenBelgium

**Keywords:** carbon retention curve, carbon sequestration, cascading, climate change, first‐order decay, half‐life, low‐carbon economy, mitigation, wood industry

## Abstract

In addition to forest ecosystems, wood products are carbon pools that can be strategically managed to mitigate climate change. Wood product models (WPMs) simulating the carbon balance of wood production, use and end of life can complement forest growth models to evaluate the mitigation potential of the forest sector as a whole. WPMs can be used to compare scenarios of product use and explore mitigation strategies. A considerable number of WPMs have been developed in the last three decades, but there is no review available analysing their functionality and performance. This study analyses and compares 41 WPMs. One surprising initial result was that we discovered the erroneous implementation of a few concepts and assumptions in some of the models. We further described and compared the models using six model characteristics (*bucking allocation*,* industrial processes*,* carbon pools*,* product removal*,* recycling* and *substitution effects)* and three model‐use characteristics (*system boundaries*,* model initialization* and *evaluation of results*). Using a set of indicators based on the model characteristics, we classified models using a hierarchical clustering technique and differentiated them according to their increasing degrees of complexity and varying levels of user support. For purposes of simulating carbon stock in wood products, models with a simple structure may be sufficient, but to compare climate change mitigation options, complex models are needed. The number of models has increased substantially over the last ten years, introducing more diversity and accuracy. Calculation of substitution effects and recycling has also become more prominent. However, the lack of data is still an important constraint for a more realistic estimation of carbon stocks and fluxes. Therefore, if the sector wants to demonstrate the environmental quality of its products, it should make it a priority to provide reliable life cycle inventory data, particularly regarding aspects of time and location.

## Introduction

According to the Fifth Assessment Report of the Intergovernmental Panel on Climate Change (IPCC, 2013), the atmospheric concentration of carbon dioxide (CO_2_), the main greenhouse gas responsible for climate change, has been increasing since the start of the Industrial Revolution. Forests' capability to sequester and store carbon was first recognized by an official body at the United Nations Framework Convention on Climate Change (UNFCCC) in 1992, and the first commitment period of the Kyoto Protocol (2008–2012) encouraged Annex I Parties to monitor and report carbon stock changes in forests. At the 17th Conference of the Parties in Durban (COP17) in 2011, harvested wood products were accepted as accounted carbon pools and thus have to be reported by all Parties included in Annex I (43 countries), and by Parties in Annex II (154 countries) on a voluntary basis. Consequently, reporting carbon stock changes is the biggest reason for new demand of wood product models.

Process‐based forest growth models estimate atmospheric carbon sequestration and allocate it to pools, such as vegetation, dead wood or soil (Peltoniemi *et al*., 2006; Fang *et al*., 2007; Garcia *et al*., 2010; Wiesmeier *et al*., 2012). Some of these models include forest management operations and estimate the amount of harvested wood (Fang *et al*., 2007; Waterworth & Richards, 2008). Humans harvest wood for very different purposes, from direct incineration of wood fuel for energy to long‐lasting construction materials. Wood product models use the allocation of harvested carbon for different purposes to estimate carbon input into different wood product classes and to evaluate how that input evolves. An earlier study using wood product models concluded that in Europe (EU15), carbon stored in wood products represents 1% of the total greenhouse gas inventory (Kohlmaier *et al*., 2007). Other studies have reported that across the entire forestry sector, including ecosystems and wood products, the cumulative carbon stored in wood products plays a significant role: 16% in the UK (Dewar & Cannell, 1992), 13% in Canada (Kurz *et al*., 1992), between 12% (Pussinen *et al*., 1997) and 4% (Eggers, 2002; Karjalainen *et al*., 2003) in Finland, 5% in the USA (Smith *et al*., 2004), 6% in Europe (Eggers, 2002; Karjalainen *et al*., 2003) and 7% in France (Fortin *et al*., 2014).

Wood product models are also used to estimate the greenhouse gas emissions derived from wood product use. Biogenic emissions are estimated though carbon stock changes. If the carbon stock increases, the wood product pool acts as a carbon sink; otherwise it acts as a carbon source. Decomposition of wood can occur under different conditions with consequences for the type of gas that is released. Decomposition of wood under aerobic conditions produces CO_2_ emissions, while under anaerobic conditions it produces non‐CO_2_ emissions such as CH_4_. Non‐CO_2_ greenhouse gas emissions are transformed to CO_2_ equivalents to harmonize calculations of the overall global warming potential.

In addition to biogenic emissions, some wood product models can estimate fossil fuel emissions from activities throughout the product life cycle, to include the full climate impact of wood product use. Life cycle inventories provide harmonized emission values using CO_2_ equivalents of all involved processes. Equally important are emissions from industries producing alternatives for wood products. While reviewing 21 international studies on wood product substitution, Sathre and O'Connor (Sathre & O'Connor, 2010a) estimated an average reduction of emissions of 2.1 tons of carbon for each ton of carbon in wood products used instead of alternative products.

Climate change effects on carbon storage in wood products have been only cursorily analysed. Studies by Karjalainen and others (Karjalainen *et al*., 2002, 2003) have predicted carbon stock increases in the European forestry sector as a whole due to a predicted increase in net primary production. However, a predicted increase in natural disturbances should also be included when estimating future carbon stocks in wood products (Cameron *et al*., 2013) by the representation of an increasing annual variability of the amount of harvested wood. Fortin *et al*. (2014) estimated that by omitting windstorm damages, the carbon stock in wood products could be overestimated by as much as 8%.

When wood product models are linked with forest ecosystem models, researchers can compare forest management strategies and alternative product uses to maximize climate change mitigation effects of the forestry sector as a whole. Lemprière *et al*. (2013) described this overall approach as a “system perspective” and it has been implemented, for example, for Canada using the NFCMARS version of the WPM reviewed here (Smyth *et al*., 2014). Silvicultural recommendations may include species (Pukkala, 2011), tree density (Fortin *et al*., 2012), thinning type (Profft *et al*., 2009; Pukkala, 2014), forest canopy (Thornley & Cannell, 2000) or rotation lengths (Liski *et al*., 2001; Kaipainen *et al*., 2004; Perez‐Garcia *et al*., 2005; Pingoud *et al*., 2010). Recommendations in the wood sector focus on the allocation of harvested wood to long‐lasting products (Harmon *et al*., 1996; Werner *et al*., 2006; Eriksson *et al*., 2007; Fortin *et al*., 2012; Smyth *et al*., 2014) and to products with high recycling rates (Werner *et al*., 2010; Klein *et al*., 2013).

Different assumptions may result in contradictory conclusions. For example, conclusions on rotation length are affected by whether or not a bucking allocation module is included. This module assigns grades to harvested wood used in different industries according to log characteristics. Studies including a bucking allocation module recommend long rotations for maximizing carbon stocks in the forest sector as a whole (Liski *et al*., 2001; Pingoud *et al*., 2010), while studies excluding it recommend short rotations (Kaipainen *et al*., 2004; Perez‐Garcia *et al*., 2005). All assumptions should be reviewed when comparing or using results from different studies, so as to avoid incomparable conclusions.

A review describing and comparing wood product models is missing. A description of model elements and assumptions would help future researchers to select a suitable model based on better knowledge and addressing the requirements of the purpose of the study. It would also foster further model improvement, allowing for a clearer focus on the overall knowledge gaps existing in the modelling platform and helping researchers to avoid common errors. With this review, we aim to close this gap by describing model characteristics in a comparative way and by identifying misused concepts and assumptions. We have avoided mentioning the specific models misusing concepts or assumptions because we simply wanted to raise awareness of their correct use. By doing so, we can avoid confusing readers who might interpret us as saying that only the models mentioned misuse these concepts and assumptions. Next, we classify existing models and analyse their evolution. Finally, we propose possible future improvements to achieve more accurate results.

## Materials and methods

First, we analysed the model characteristics of 41 published wood product models (Table 1). We found these wood product models using online scientific citation indexing service tools (*Web of Science* and *Scopus*). Six of the models had two or three versions; these were analysed independently of one another. To interpret the models and their characteristics in a correct way, we contacted the authors of the articles or the model developers when available to obtain more background information.

**Table 1:** Wood product models and references used as information sources

Model name or first author surname	Year	References	Abbreviation
Terrestrial Carbon Model	1983	Houghton *et al*. (1983); R.A. Houghton Personal communication	TCM
CARBINE	1989	Thompson & Matthews (1989a,b)	CARBINE
FORPROD (1st version)	1990	Harmon *et al*. (1990)	FORPRO1
Dewar	1992	Dewar (1990, 1991); Dewar & Cannell (1992)	Dewar
HARVCARB	1991	Row & Phelps (1991); Plantinga & Birdsey (1993); Heath *et al*. (1996); Row & Phelps (1996)	HARVCAR
CBM‐FPS (1st version)	1992	Kurz *et al*. (1992)	CBMFPS1
Karjalainen	1994	Karjalainen *et al*. (1994)	Karjala
FORCARB (1st version)	1995	Birdsey & Heath (1995)	FORCAR1
FORPROD (2nd version)	1996	Harmon *et al*. (1996); M.E. Harmon Personal communication	FORPRO2
GORCAM	1996	Schlamadinger & Marland (1996)	GORCAM
Winjum	1998	Winjum *et al*. (1998)	Winjum
CBM‐FPS (2nd version)	1999	Apps *et al*. (1999)	CBMFPS2
CO2FIX (version 1.2)	1999	Mohren & Klein Goldewijk (1990); Mohren *et al*. (1999)	CO2FIX1
Edinburgh Forest Model	2000	Thornley & Cannell (2000)	Edinbu
CO2FIX (version 2.0)	2001	Nabuurs *et al*. (2001); Masera *et al*. (2003)	CO2FIX2
EFISCEN	2002	Eggers (2002), H. Verkerk Personal communication	EFISCEN
CO2FIX (version 3.1)	2004	Schelhaas *et al*. (2004); G.J. Nabuurs Personal communication	CO2FIX3
XYLOIKOS Model	2004	Muller *et al*. (2004)	XYLOIKO
FORCARB2	2004	Heath *et al*. (2003); Smith *et al*. (2004, 2006)	FORCAR2
White	2005	White *et al*. (2005)	White
Perez‐Garcia	2005	Perez‐Garcia *et al*. (2005)	Perez‐G
Dias (1st version)	2005	Dias *et al*. (2005)	Dias1
Werner (1st version)	2006	Werner *et al*. (2006)	Werne1
Green	2006	Green *et al*. (2006)	Green
IPCC HWP	2006	IPCC (2006)	HWPIPCC
CAMFor	2007	Richards *et al*. (2007); D. Evans Personal communication	CAMFor
Frankfurt HWP model	2007	Kohlmaier *et al*. (2007)	Frankfu
Dias (2nd version)	2007	Dias *et al*. (2007, 2009); A. Dias Personal communication	Dias
Seidl	2007	Seidl *et al*. (2007)	Seidl
Eriksson	2007	Eriksson *et al*. (2007)	Erikss
Carbon Object Tracker (CO_T_)	2008	Hennigar *et al*. (2008); Cameron *et al*. (2013); C. Hennigar Personal communication	COT
WOODCARB II	2008	Skog (2008); K. Skog Personal communication	WOODCII
FORCARB‐ON	2008	Chen *et al*. (2008)	FORC‐ON
Profft	2009	Profft *et al*. (2009)	Profft
CBM‐FPS (3rd version)	2009	Kurz *et al*. (2009); W.A. Kurz and M. Magnan Personal communication	CBMFPS3
Pingoud	2010	Pingoud *et al*. (2010)	Pingoud
Werner (2nd version)	2010	Werner *et al*. (2010)	Werne2
NFCMARS	2011	Stinson *et al*. (2011); M. Magnan Personal communication	NFCMARS
C‐HWP model	2011	Rüter (2011); S. Rüter Personal communication	C‐HWP
Pukkala	2011	Pukkala (2011); (Pukkala, 2014); T. Pukkala Personal communication	Pukkala
CAPSIS	2012	Fortin *et al*. (2012, 2014): M. Fortin Personal communication	CAPSIS
WoodCarb Ireland model	2012	Donlan *et al*. (2012)	WoodCar
LANDCARB	2012	Krankina *et al*. (2012); http://landcarb.forestry.oregonstate.edu/summary.aspx	LANDCAR
BC‐HWPv1	2012	Dymond (2012); M. Magnan Personal communication	BC‐HWP
Earles	2012	Mason Earles *et al*. (2012)	Earles
Klein	2013	Klein *et al*. (2013); D. Klein Personal communication	Klein
PRESTO	2014	Hoover *et al*. (2014)	PRESTO
Pilli	2015	Pilli *et al*. (2015)	Pilli
Höglmeier	2015	Höglmeier *et al*. (2015)	Höglmei

Wood product model and model‐use characteristics were identified analysing all models. They are described following the logical order of a wood product's life cycle (Table 2). The characteristics represent the most important decisions that model developers or users need to make when creating or applying wood product models. These decisions will affect the outcome in terms of carbon stocks and fluxes. For various reasons, a number of models do not include some of these features, such as bucking, recycled products or substitution effects. Due to the demand of reporting carbon stock changes in wood products following the 2013 Revised Supplementary Methods and Good Practice Guidelines (IPCC, 2014), we described the characteristics of the Tier 2 methodology corresponding to each section.

**Table 2:** Characteristics used to analyse wood product models following the logical order of a wood product's life cycle

Characteristics	Name of characteristics
Model characteristics	Bucking allocation
	Industrial processes
	Carbon pools
	Product removal
	Recycling
	Substitution effects
Model‐use characteristics	System boundaries
	Model initialisation
	Results evaluation

Evaluation and classification of models was carried out by evaluating two components: representation of reality (component *structure*) and user‐friendliness (component *use*). Each component was dealt with formulating a principle with its criteria and indicators (Table 3). In total, we defined 13 indicators under three criteria and two principles. Indicators were assigned a binary score, with 1 for compliance with a criterion and 0 for noncompliance. The threshold for compliance or noncompliance of each criterion is defined in the description column of Table 3. The percentage of indicators that scored 1 was calculated per component. High ratings for the *structure* component indicate models with high complexity and close to reality. High ratings for the *use* component point to more user‐friendly models. The indicators for the *structure* component are based on the modelling characteristics identified previously, with a few adaptations. *Product removal* was excluded because current data on product removal is ambiguous and we were unable to identify which distributions are closer to reality. The criterion *carbon pools* was subdivided into *number of pools* and *disposal site* to distinguish products in use from disposed products. *Versatility of allocation parameters* was introduced as an additional indicator to draw attention to time‐sensitive models.

**Table 3:** Hierarchical framework of principles, criteria and indicators to classify wood product models

Component	Principle	Criteria	Indicator	Description
Structure	Model structure is close to reality	Model structure reflects all relevant processes	Bucking allocation module	Does the model include a bucking allocation module?
			Industrial processes	Are industrial processes reflected in the model?
			Number of pools	Does the model include more than three carbon pools of products in use as recommended by the IPCC guidelines (sawn wood, wood based panels, and paper and paperboard)?
			Disposal site	Does the model include pools after disposal?
			Recycling	Does the model include recycling?
			Substitution effect	Does the model consider material or energy substitution?
		Model structure is versatile regarding industrial changes	Versatility of allocation parameters	Does the model allow parameter changes over time?
Use	Model is user‐friendly	Model is easily understood and applied by external users	Available interface	Does the model have an interface?
			Code transparency	Can users get access to the code?
			Training opportunities	Is training to use the model organised?
			Technical support service	Is technical support service provided?
			User community	Does a user community exist?
			Updates	Is the model being updated?

The indicators for the *use* component are not based on model‐use characteristics because model‐use characteristics do not evaluate the model itself, but how it is applied. The same model can be applied differently, for instance, with different system boundaries. Thus, indicators for the *use* component were modified substantially in comparison to the model‐use characteristics to better describe the models themselves, instead of their application (see Tables 2 and 3).

An initial model evaluation was performed using information available in the literature. Then, we asked model authors to check our evaluation and to complete and correct if necessary. In total, we sent 49 e‐mails and received 23 answers referring to 16 models (39% of the total).

The final evaluation score for each indicator was entered into *RStudio* software (version 0.98.501) to compute a dissimilarity matrix (*vegdist* in *vegan* package, version 1.16‐32 for *R*, using the *Euclidean* method) as the input for a hierarchical cluster analysis (*hclust* in *stats* package, version 2.15.3 for *R*, using the *complete* method). From the clustering results, we identified the greatest height difference in the dendrogram, and counted the amount of clusters at that level. This methodology will allow future model users to undertake an initial selection of models according to the objectives of their studies and their experience in using wood product models.

## Wood product model characteristics

### Model characteristics

#### Bucking allocation

Bucking allocation refers to the allocation of logs to harvested products (e.g. roundwood, pulpwood or slash wood). Species, wood quality and stem diameter are the main factors determining wood allocation. Some models include this in the parameters of industrial processes (see the next characteristic), but this wood allocation strategy only considers average tree characteristics and may cause errors when, for instance, evaluating the effect of modifying rotation length. Models using official statistics on product types, like models following the Tier 2 methodology recommended by the IPCC, do not need the bucking allocation module, because the products are already categorized.

#### Industrial processes

Wood product models allocate carbon from harvested products to products in use via processes of primary (e.g. sawmills or wood‐based panel producers) and secondary (e.g. construction, furniture or packaging) wood processing industries, paper industries and energy industries. When products arrive at their end of use, they may be disposed of or recycled. Industrial processes, recycling and disposal define the allocation parameters used in each transformation step. Some models allow these parameters to change over time to account for technical improvements or behavioural changes.

We identified two types of models according to the way they present industrial processes. The first type sees industrial production as an input, so industrial processes are not represented. The IPCC Tier 2 methodology recommended applying this approach. Models like C‐HWP (Rüter, 2011) or the Frankfurt Harvested Wood Products model (Kohlmaier *et al*., 2007) use this approach, employing FAOSTAT data as a source to quantify production amounts for different product categories. The second model type uses harvested wood as the input, and industrial processes are represented by allocation parameters. Examples of this group are models used in Profft *et al*. (2009) or Eriksson *et al*. (2007). In this case, information on allocation parameters comes from expert knowledge, industry surveys or life cycle inventories, but the use of parameters from previous studies is a common practice.

At the end of its life, wood may be recycled, disposed of in landfills or dumps, or burned, with or without energy production. The share of wood product waste for each of these fates depends on product type, time and location. A lack of reliable information sources regarding the end of life of wood products is typical, with paper products being an exception.

#### Carbon pools

Carbon pools in wood product models are represented by wood products in use and in disposal sites. Paper products are considered as wood products in this article, as in most of the literature (Smith *et al*., 2006).

Wood products in use are characterized by their capacity to store carbon over an average time. Some models like CAPSIS (Fortin *et al*., 2012), WOODCARB II (Skog, 2008) or BC‐HWPv1 (Dymond, 2012) define pools by their utility, e.g. *paper*,* packaging*,* furniture* or *construction wood*. The Decision 2/CMP.7 Tier 2 method requires default half‐lives for three product categories, also defined by their utility (IPCC, 2014): sawn wood (35 years), wood‐based panels (25 years), and paper and paperboard (2 years). Other models, such as EFISCEN (Karjalainen *et al*., 1994; Eggers, 2002), GORCAM (Schlamadinger & Marland, 1996) or CO2FIX (Schelhaas *et al*., 2004) define pools by comparative lifespans, i.e. *short*,* medium* and *long*. In practice, there is no difference between the systems, since each pool gathers different products with similar lifespans. Pingoud *et al*. (2003) provided a list of lifespan values used in the literature.

Carbon pools in disposal sites are distinguished by the presence or absence of oxygen during wood decomposition. In open dumps, oxygen is available and wood decomposes completely. Landfills, however, are sealed, and the lack of oxygen creates different conditions for the decay of the major polymeric components of wood (cellulose, hemicellulose and lignin). During anaerobic decomposition, microbial activity decomposes the portion of degradable organic carbon (cellulose and hemicellulose) to methane and CO_2_ (Micales & Skog, 1997; Barlaz, 2006). Lignin, on the other hand, is considered recalcitrant (Colberg, 1988). The 2006 IPCC Guidelines (IPCC, 2006) recommended using 50% as the fraction of degradable organic carbon, but later laboratory (Wang *et al*., 2011) and field studies (Wang *et al*., 2013) have shown that the fraction of degradable organic carbon is below 50%. Nevertheless, in the 2013 Revised Supplementary Methods (IPCC, 2014), CO_2_ emissions due to decomposition of carbon stock in wood products being landfilled is either reported as oxidation or with a time delay by the wood products sector. Instead, non‐CO_2_ emissions are reported by the waste sector.

Taking into account that carbon is stored in landfills for many years, landfills are still important carbon stocks, even in Europe where landfilling has not been allowed since the Landfill Directive (1999/31/EC). Due to this prohibition, some European models exclude landfills. Most of the models including landfills only estimate the carbon stock. However, the global warming potential of landfill emissions changes depending on the type of gas emitted. Therefore, simply estimating the carbon stock change in landfills is inadequate when aiming to estimate total greenhouse gas emissions. Models should distinguish between gases, and then calculate the CO_2_ equivalent. Some models already distinguish gases from landfill emissions (e.g. CBM‐FPS, CO2FIX, NFCMARS and BC‐HWP).

#### Product removal

Product removal refers to the point in time when products are retired from use. Removal of products depends on many factors, including not only their functional lifespan, but also economic cycles or fashion trends. This means that products with identical characteristics might be retired at different moments in time, depending on the spatio‐temporal context of their use. However, the main problem in estimating product removal continues to be a lack of data.

Wood product models estimate the removal rate using carbon retention curves. These curves are defined by a chosen statistical distribution and by the time after production when a certain percentage of the product remains in use. Thompson & Matthews (1989a,b) were pioneers in defining carbon retention curves. They used a Weibull distribution; for each product category, they estimated when carbon loss was at its maximum rate, and when 5% of the initial amount of carbon remained. The parameterization of these curves was based on expert judgements by Donald Thompson, who at that time was the British Forestry Commission's Wood Utilization Officer (R. Matthews, Personal communication). Later studies (Kurz *et al*., 1992; Karjalainen *et al*., 1994; Harmon *et al*., 1996; Smith *et al*., 2006) published new carbon retention curves, also based on expert judgements from different jurisdictions/countries, but applying different distribution functions.

Statistical distributions applied in the literature have included uniform (Pingoud *et al*., 2003), linear (Winjum *et al*., 1998), Weibull (which includes exponential distribution) (Karjalainen *et al*., 1994), logistic (Eggers, 2002), normal (Muller *et al*., 2004) and gamma (Klein *et al*., 2013) distributions. Each distribution is defined by one or two of the following descriptors defining the years after production: median or 50% of carbon left (also known as half‐life), 5% of carbon left, mean or average life (also called mean residence time), and mode or time at maximum rate of carbon loss. Some distributions used other parameters, but were based on the previous ones: e.g. linear distribution uses a constant annual oxidation (year^−1^), normal distribution uses mean and standard deviation, and gamma distribution uses shape and scale. The selection of a distribution function may have a substantial effect on the resulting carbon stock calculations, as illustrated in the simulation exercise in S1.

In addition to lifespan and distribution functions, wood product models may use two other approaches to estimate carbon removal: the single pool approach and the distributed approach. The single pool approach assumes only one pool for each product category; as a result, product removal is affected by the total amount of stock, but not by product age. In this case, carbon stock change is estimated as a fraction of production, and production is assumed to be exponentially increasing (Marland *et al*., 2010). On the other hand, the distributed approach considers as many pools as production years for each product category. In this case, the rate of removal depends on product age instead of the amount of stock (Marland *et al*., 2010).

The first‐order decay approach was recommended by the 2006 IPCC Guidelines (2006) Tier 1 and 2, and again by Tier 2 of the 2013 Revised Supplementary Methods (IPCC, 2014). The first‐order decay approach uses the exponential decay function.

#### Recycling

At the end of use, wood products may also be collected and transformed into new products to be recycled. A cascade chain defines the steps that a wood fibre may go through before being disposed of or burned. The idea of cascading was developed with the aim of reducing the appropriation of net primary production of ecosystems by increasing harvested wood efficiency and maximizing its socio‐economic advantages (Haberl & Geissler, 2000). One example of a cascade chain recommended by Sikkema *et al*. (2013) would be to use high‐quality logs for sawn wood first, for panel production afterwards, and finally combusted with energy recovery. In the case of paper, fibres can be recycled four or even up to six times before the fibre length becomes too short (Schmidt *et al*., 2007).

Some wood product models simply exclude recycled products altogether. Some other models using official databases, like the ones following the IPCC Tier 2 methodology, include recycled products as inputs. Other models include recycling as a percentage of removed product type which is sent to other product categories. For instance, the CO2FIX default recycling parameter for long‐lasting products is 30%, from which 10% is sent to long‐lasting products, 30% to medium‐lived products, and 60% to short‐lived products (Schelhaas *et al*., 2004). However, only one model includes recycling percentages using predefined cascade chains (Höglmeier *et al*., 2015). The practice of using recycling rates instead of defined cascade chains leads to errors. For example, if 100 kg of paper is produced, assuming a recycling rate of 70% for paper, 70 kg will be recycled in the first round, 49 in the second, 34.3 in the third, then 24.1, then 16.8, and in the sixth round 11.8 kg. However, this 11.8 kg of recycled paper can no longer be recycled (see above), and this restriction is not included in any model. Other products such as boards, with other quality requirements concerning raw materials, may face similar problems. Particle board is of lower quality when panel particles are steam‐recovered when compared to particle board from virgin particles (Lykidis & Grigoriou, 2008). It is important to be aware of biomass quality requirements when producing a new product from waste wood (Haberl & Geissler, 2000).

Cascade practice has been increasing since the recovery of construction wood from demolition sites has become more widely implemented (McKeever, 2004). Cascading of biomass may have mitigation benefits due to a reduction of CO_2_ emissions (Dornburg & Faaij, 2005). Reused paper reduces greenhouse gas emissions considerably after one recycling round (Sikkema *et al*., 2013). For other products, such as oriented strand board (OSB), utilization of waste wood is attractive as a way to reduce greenhouse gas emissions, but this cascade step is relatively underdeveloped (Sikkema *et al*., 2013). Nevertheless, mitigation effects of cascading depend on the CO_2_ emissions of the reference system. In fact, emissions surrounding waste wood collection, transport and production of a new product could even imply an increase in production emissions when compared to alternative materials (Dornburg & Faaij, 2005). As a consequence, short cascade chains like pellet and energy production with high emission reductions may sometimes turn out more favourably when compared to long cascade chains (Dornburg & Faaij, 2005).

#### Substitution effects

The life cycle inventories of a functional unit (e.g. one house, one m^3^ of sawn wood or one J of energy produced) estimate greenhouse gas emissions during material extraction, industrial transformation, use phase and disposal. Data extracted from these inventories may be included in models and used to estimate emitted greenhouse gases due to wood product use. With this aim, models must estimate the number of functional units produced. Materials with high energy requirements for production might be substituted by less energy‐demanding wood products. Mitigation effects from using wood products can thus be obtained by reducing greenhouse gas emissions from competitive industrial sectors when wood replaces other materials satisfying the same function. Such a reduction of greenhouse gas emissions as a consequence of product substitution is called a substitution effect, and it is important due to the fact that these avoided emissions are permanent and cumulative (Eriksson *et al*., 2007). However, the benefits of substitution are not reported to the UNFCCC within the Land Use Land‐Use Change and Forestry sector as the emission reductions will be captured in other sectors (and in other countries). Industrial emissions are reported by the industrial sector. Substitution effects in turn may lead to a climate change mitigation effect that might be larger than carbon storage (Schlamadinger & Marland, 1996; Werner *et al*., 2010), which is not permanent. Substitution effects include material substitution (or indirect substitution) referring to the replacement of materials such as steel or concrete, and energy substitution (or direct substitution) referring to replacement of fossil fuels like oil or gas.

Gustavsson *et al*. (2006b) found that the production of wood‐framed buildings in Scandinavian countries requires less energy and emits less CO_2_ than the production of functionally equal concrete‐framed buildings. Moreover, during the use phase, wood‐based houses require less heating and cooling energy than houses with comparable thermal insulation constructed using alternative materials such as steel or concrete (Upton *et al*., 2008). Results of analysis of climate change mitigation effects of using wood products are affected by the inclusion of substitution effects (Perez‐Garcia *et al*., 2005; Hennigar *et al*., 2008). Wood utilization produces fewer emissions and less waste, but preservative‐treated wood might have toxicological impacts on human health and ecosystems when burned (Petersen & Solberg, 2005).

The analysis of wood substitution effects is complex, including several industries, socio‐economic and cultural aspects, traditions, cost dynamics, technical and structural changes, and so on (Gustavsson *et al*., 2006a). Studies such as those by Petersen & Solberg (2005) and Werner *et al*. (2006) regarding materials, or by Petersen (2006) regarding energy have used life cycle assessments to estimate substitution effects. Displacement factors or emission factors have been used to estimate substitution effects in, e.g. Eriksson *et al*. (2007), Werner *et al*. (2010), Sathre & O'Connor (2010a) and Helin *et al*. (2013). These factors depend on a reference scenario that may change by location and over time due to differences in the reference fuel or technological developments (Pingoud *et al*., 2010). In most cases, the displacement factor is positive, meaning that more emissions are avoided than caused when using wood materials instead of alternative materials. However, it could be negative, for example in the case of substituting conventional print media by web‐based media (Pingoud *et al*., 2010). Klein *et al*. (2013) conducted a literature review about displacement factors for substitution of materials, while Sathre & O'Connor (2010b) undertook one for material and energy substitution. When wood replaces other energy sources, fossil fuels (including oil, coal and natural gas) have been the only alternatives so far. However, according to Eurostat (2014), the proportion of renewable energy sources is currently increasing and should therefore be included when estimating emission factors.

### Model‐use characteristics

#### System boundaries

The 2006 IPCC Guidelines (IPCC, 2006) described four approaches to defining system boundaries for wood product models. Although these approaches were designed for use at national levels, these can be applied at subnational or local levels as well. The selected approach depends on the case study considered and not on the model, but we consider it relevant to briefly describe them.

- The *Stock‐Change Approach* estimates carbon stock changes in forests and wood product pools physically located in the studied region. Forests growing and products used in the region of study are reported. Exported wood and forests growing outside of the study region are not considered, but emissions from imported wood are included.
- The *Atmospheric Flow Approach* estimates carbon stored in wood products consumed in the region and from local forests. Local forests producing products exported and consumed in other regions are counted, but the emissions from the exported products are not counted.
- The *Production Approach* estimates carbon from regional forests and their products. Carbon stock in exported products is considered, but carbon stock in imported products is not. Most analysed studies used this approach. Emissions from exported products are counted.
- The *Simple Decay Approach* estimates carbon stored in wood products consumed in the region and from local forests. Local forests producing products exported and consumed in other regions are counted as well as the emissions from the exported products.

Several authors have compared the effect of applying different approaches on the same region. For example, Kohlmaier *et al*. (2007) conducted a comparison between the Stock Change and the Production Approaches in the EU15 community, and estimated carbon stored in products in use to be 10.83 Mt C/a based on the Stock‐Change Approach and 9.81 Mt C/a based on the Production Approach.

#### Model initialization

Model initialization aims at estimating values for each state variable for the first year of the model run. In some cases, initialization is omitted; for example, Fortin *et al*. (2012) did not initialize the model because they expressed the results on an average basis, integrating the carbon stocks over the rotation period and the lifetime of the harvested products. Other studies avoid initialization and simply focus on the decay rate of products to compare different scenarios.

We identified three possible strategies to initialize wood product models. The first strategy is to use inventory data from a specific year for each state variable, for instance the number of houses, the amount of wood used per house, and the amount of wood used per house restoration. This type of data might be provided by independent scientific studies or national census bureaus, using different methods. One disadvantage of this option is the general lack of inventories; moreover, when they are available, they do not cover all product types. Another disadvantage is that the product age is unknown, and therefore the remaining lifespan is also unknown. We found no studies using this method due to the poor quality of the data available, though Richards *et al*. (2007) and Skog (2008) both used inventories to validate their results or to calibrate parameters.

The second strategy is to run the model for a long enough period of time using data on wood production from official databases like EUROSTAT or FAOSTAT. The IPCC recommended this strategy. These sources need to cover long periods to ensure that the carbon sequestered during the initial years of the study has been emitted back into the atmosphere after its use, recycling and decomposition phases. In the literature under review, we found different lengths of time for this. The 2013 Revised Supplementary Methods (IPCC, 2014) recommended using harvested products since the year 1900 (extrapolating the averaged earliest 5 years of available data when earliest data is not available), arguing that effects of earlier harvested products are insignificant. Lippke *et al*. (2011) suggested a minimum of approximately 80 years. Thompson & Matthews (1989a,b) estimated different values between species, from 45 years for birch up to 150 for oak, excluding recycling effects. Green *et al*. (2006) used 42 years of data from the FAOSTAT database (from 1961 to 2003). On the other hand, Karjalainen *et al*. (2002), also using the FAOSTAT database, were concerned about having a data series that was too short, and extrapolated 1961 data to the period of 1931–1960, and eventually had a prerun of 60 years (1931–1990). Notice that this presimulation is not aiming to approximate steady‐state conditions (since input data oscillates), but instead to estimate carbon stock on the first year of simulation and to be able to analyse carbon stock changes.

The third strategy is similar to the second one, but the prerun uses inputs from forest growth models. Both models, the forest growth model and the wood products model, are run for long periods until the state variables become stabilized, as Muller *et al*. (2004) or Pingoud *et al*. (2010) did. This spin‐up simulation approximates steady‐state conditions.

#### Results evaluation

Once models have been transferred onto computers, the developer needs to check model stability in the long run, and if it behaves as expected. According to modelling theory (Jørgensen & Fath, 2011), model users should follow a few steps when they want to apply a model. These steps include sensitivity analysis, calibration, validation and uncertainty analysis. The goal of the sensitivity analysis is to gain an overview of which parameters have stronger impacts on results. Calibration aims to improve the estimates of all parameters. Validation compares how close to reality model results are. Finally, the uncertainty analysis evaluates how certain the user can be regarding the obtained outcome. In those cases when results are sensitive to a particular input variable and this variable is uncertain, the results could also be uncertain and therefore the user should invest more efforts in reducing variable uncertainity.

The IPCC guidelines defined it as a good practice to identify, quantify and reduce the uncertainities as much as practicable. However, wood product models are weak regarding these modelling components. Sensitivity analysis was applied to 48% of the analysed models, uncertainty analysis to 40% of them, calibration to 19%, and validation to only 15%. These modelling components are often not applied in wood product models because data sources are difficult to obtain (e.g. data from wood‐based industries), or because no official estimation exists (e.g. data on the lifespan of final products). When modelling activities such as validation and calibration are not completed during model application, results are based on many assumptions; therefore, final conclusions could be inaccurate.

## Wood product model classification

**Figure 1:**
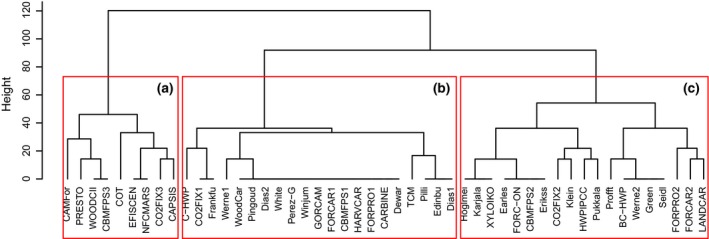
Dendrogram showing the result of the wood product model classification. The three boxes indicate the three Groups (A, B and C), distinguished by the largest dissimilarity. Group A gathers models having high scores on *structure* and *use* components. Group B includes models having low scores on these components. Group C gathers models having high scores on *structure* component and low scores on *use* component.

We identified three main groups of models from the clustering dendrogram (Table 4, Fig. 1). The first group (A) includes models with complex structures and support for easy use. Models in the second group (B) are characterized by a simple structure but no support for ease of use. The third group (C) gathers models having a complex structure, but little help for their utilization.

**Table 4:** Results of wood product model evaluation and classification. All indicators are binary, with value 0 for absence and 1 for presence (for a definition of binary values, see Table 3)

Model	Buc	Ind	Poo	Dis	Rec	Sub	All	STR (%)	Int	Cod	Tra	Sup	Com	Upd	USE (%)	Group
TCM	0	0	0	0	0	0	0	0.0	0	0	0	0	0	1	16.7	B
CARBINE	1	0	1	0	0	0	0	28.6	0	0	0	0	0	0	0.0	B
Dewar	1	0	1	0	0	0	0	28.6	0	0	0	0	0	0	0.0	B
FORPRO1	0	1	0	0	1	0	0	28.6	0	0	0	0	0	0	0.0	B
HARVCAR	0	1	1	0	0	0	0	28.6	0	0	0	0	0	0	0.0	B
CBMFPS1	0	1	0	1	0	0	0	28.6	0	0	0	0	0	0	0.0	B
Karjala	0	1	1	1	1	0	0	57.1	0	0	0	0	0	0	0.0	C
FORCAR1	0	0	0	1	1	0	0	28.6	0	0	0	0	0	0	0.0	B
GORCAM	0	1	0	0	0	1	0	28.6	0	0	0	0	0	0	0.0	B
FORPRO2	1	1	1	1	1	0	1	85.7	1	1	0	0	0	0	33.3	C
Winjum	0	1	1	0	0	0	0	28.6	0	0	0	0	0	0	0.0	B
CBMFPS2	0	1	0	1	1	0	0	42.9	0	0	0	0	0	0	0.0	C
CO2FIX1	0	1	0	0	1	0	0	28.6	1	0	0	0	0	0	16.7	B
CBMFPS3	0	1	1	1	1	1	1	85.7	0	0	1	1	0	1	50.0	A
Edinbu	0	0	0	0	0	0	0	0.0	0	0	0	0	0	0	0.0	B
CO2FIX2	0	1	0	1	1	0	0	42.9	1	0	0	0	0	0	16.7	C
EFISCEN	0	1	1	1	1	1	0	71.4	1	1	1	1	0	0	66.7	A
FORCAR2	0	1	1	1	1	1	0	71.4	0	1	0	0	0	0	16.7	C
XYLOIKO	0	1	0	0	1	1	1	57.1	0	0	0	0	0	0	0.0	C
CO2FIX3	0	1	1	1	1	1	0	71.4	1	1	1	1	1	0	83.3	A
Perez‐G	0	1	0	0	0	1	0	28.6	0	0	0	0	0	0	0.0	B
White	0	1	1	0	0	0	0	28.6	0	0	0	0	0	0	0.0	B
Dias1	0	0	0	0	0	0	0	0.0	0	0	0	0	0	0	0.0	B
HWPIPCC	0	0	1	1	0	1	0	42.9	1	1	0	0	0	0	33.3	C
Werne1	0	0	0	0	0	1	0	14.3	0	0	0	0	0	0	0.0	B
Green	1	1	1	1	1	0	0	71.4	0	0	0	0	0	0	0.0	C
CAMFor	0	1	1	1	1	0	0	57.1	1	1	1	0	0	0	50.0	A
Frankfu	0	0	1	1	0	0	0	28.6	1	0	0	0	0	0	16.7	B
Erikss	0	1	0	0	1	1	0	42.9	0	0	0	0	0	0	0.0	C
Dias2	1	0	1	0	0	0	0	28.6	0	0	0	0	0	0	0.0	B
Seidl	0	1	1	1	1	1	0	71.4	0	0	0	0	0	0	0.0	C
WOODCII	1	1	1	1	1	0	1	85.7	0	1	1	0	0	1	50.0	A
FORC‐ON	0	1	1	0	1	0	0	42.9	0	0	0	0	0	0	0.0	C
COT	1	1	1	1	1	1	1	100.0	1	0	1	1	0	1	66.7	A
Profft	1	1	1	1	1	1	0	85.7	0	0	0	0	0	0	0.0	C
Pingoud	0	1	0	0	0	1	0	28.6	0	0	0	0	0	0	0.0	B
Werne2	1	1	1	0	1	1	0	71.4	0	0	0	0	0	0	0.0	C
C‐HWP	0	0	1	0	0	0	0	14.3	1	0	0	0	0	1	33.3	B
NFCMARS	0	1	1	1	0	1	1	71.4	0	1	1	1	0	1	66.7	A
Pukkala	0	1	1	0	1	1	0	57.1	1	0	0	0	0	1	33.3	C
WoodCar	0	1	1	0	0	0	0	28.6	0	0	0	0	0	0	0.0	B
CAPSIS	1	1	1	1	1	1	0	85.7	1	1	1	0	1	1	83.3	A
BC‐HWP	0	1	1	1	1	0	1	71.4	0	0	0	0	0	0	0.0	C
LANDCAR	0	1	1	1	1	1	1	85.7	1	0	0	0	0	0	16.7	C
Earles	1	0	0	1	1	0	0	42.9	0	0	0	0	0	0	0.0	C
Klein	0	1	1	0	1	1	0	57.1	0	1	0	0	0	0	16.7	C
PRESTO	1	1	0	1	1	1	0	71.4	1	1	0	1	0	0	50.0	A
Pilli	0	0	0	0	0	0	0	0.0	0	0	0	0	0	0	0.0	B
Höglmei	0	1	1	0	1	1	0	57.1	0	0	0	0	0	0	0.0	C

Model names are abbreviated according to Table 1. STR and USE columns indicate the percentage of indicators that scored 1 for the components *structure* and *use,* respectively. The column “Group” indicates to which group a model belongs as a result of the classification. Indicators are abbreviated as follows: Poo: Number of pools. Ind: Industrial processes. Buc: Bucking allocation module. Dis: Disposal site. Rec: Recycling. Sub: Substitution effect. All: Versatility of allocation parameters. Int: Available interface. Cod: Code transparency. Tra: Training opportunities. Sup: Technical support service. Com: User community. Upd: Updates.

*Structure* is the principal component of models where developers have invested more scientific efforts. On the other hand, the *use* component is only developed when a model is meant to be shared with a user community. In general, models have higher scores for the *structure* component than for the *use* component. In the *structure* component, *versatility of allocation parameters* is the indicator with the lowest score (present in 17% of all models) and the *bucking allocation module* the second lowest (present in 21% of all models). *User community* and *technical support service*, both belonging to the *use* component, are the indicators that scored below 15% for all analysed models.

Models in Group A obtained a high score for the component *structure*, although the *bucking allocation module* is present in 33% of its models, reflecting the fact that it is not required in those models that allocate harvest to product categories based on existing statistics. Moreover, having the possibility to modify allocation parameters over time is still largely missing in this advanced group of models, with only 44% of them including it. The *use* component scored well in Group A, but even so, the *user community* is only present in 22% of the models in this group.

## Model evolution

We observed an increasing interest in wood product modelling over the last ten years. The oldest model we found was published in 1983 and the newest in 2015. During the first 20 years (from 1983 to 2002), 16 models were published, including three new versions. During the last ten years or so (from 2003 to 2015), 33 models have been published, including three new versions.

The *structure* of models has become more complex with time, but recent models also make their use easy. Early models obtained low scores for both model structure and use components. On the other hand, recent models are more diverse and obtained either low or high scores for both aspects. This diversification is due to models built for different purposes. For instance, models focused on reporting to the UNFCCC have simple structures. *Substitution effect* and to a lesser extent *recycling* were not included in early models, but in recent years, as climate change mitigation has grown in political importance, these elements have been given due consideration. Interestingly, the *bucking allocation module* is not more frequent in recent models than in older ones. Finally, we consider *versatility of allocation parameters* an important indicator because it allows the inclusion of technological improvement, but its presence has only increased modestly.

We analysed different versions of six models. We found that, in all cases, the updated versions obtained higher scores. In almost all cases, the new versions of the model were classified into a more complex group due to higher scores (from Group B to Group C or from Group C to Group A). In only one case was a model classified in the same group as it was previously, though its score increased as well. Higher evaluations were mainly due to higher scores in the *structure* component, but in a few cases the *use* component improved as well.

## Discussion

The aim of this study was to review existing wood product models, and to describe their characteristics and assumptions, as well as to classify the models and map their evolution. We identified six important model characteristics to describe the models themselves (*bucking allocation*,* industrial processes*,* carbon pools*,* product removal*,* recycling* and *substitution effect*) and three regarding their use (*model initialization*,* system boundaries*, and *modelling components*). We used 13 indicators to classify the models. Model characteristics define models and their use, and affect the results. On the other hand, indicators on the *use* component do not affect the results, but evaluate how easy it is to use them. Indicators on the *structure* component evaluate how close any given model is to reality.

We identified at least five uses of wood product models: estimation of carbon stock changes, estimation of greenhouse gas emissions (with or without a distinction between CO_2_ and non‐CO_2_ greenhouse gas emissions, and between biogenic and fossil fuel emissions), estimation of the substitution effect, estimation of climate change effect on carbon stock, and estimation of the forest management effect on carbon stock in wood products. Each model has been developed with the goal of achieving one or more of these purposes. Estimation of carbon stock changes is an important purpose for models for people who need to report to the UNFCCC using the IPCC 2006 Guidelines. Some models are specifically built for this purpose following the Tier 2 methodology of the IPCC guidelines (e.g. C‐HWP). These models can be easily applied in any country using data from the FAOSTAT database. Other country‐specific models have been developed with the same purpose, but they follow the Tier 3 methodology, which requires country‐specific data (e.g. WOODCARB II or CBM‐FPS).

This review may help to avoid some misused concepts on wood product modelling. We identified a number of errors or misunderstandings shared among a few models. We hope that the definition and comparison of the approaches assumed to estimate the product removal will help future users to avoid further confusion.

In general, model descriptions do not specify if the single pool approach or the distributed pool approach is used, although in most cases the formula defining the product decay clarifies it. Confusion only appears when the product decay is defined using the rate (years^−1^). The decay defined using a rate in years^−1^ leads to confusion, because it can be used either when the model uses the single pool approach combined with the exponential decay function, or when the model uses the distributed approach combined with the linear or exponential decay function.

Other errors in wood product model application included omission of the bucking allocation module when comparing forest management effects or not defining cascade chains and using recycling loops instead. If the bucking allocation module is omitted, the statistics need to reflect the product assortment according to the different management scenario applied. The use of recycling loops overestimates carbon stocks with increasing error as the estimated recycling rate increases. Additionally, some models do not include disposal sites, but landfills have been demonstrated to be very important carbon pools, even more so than products in use. Landfilling also continues to be relevant in Europe, even many years after it was banned.

Carbon stock change cannot be straightforwardly used to estimate greenhouse gas emissions or climate change mitigation potential. As the 2013 Revised Supplementary Methods and Good Practice Guidelines (IPCC, 2014) suggest, carbon stock change in wood products in use can be estimated to identify the carbon pool effect of wood products. Forests sequester atmospheric CO_2_ to produce wood, and carbon stored in wood products is emitted as CO_2_ and CH_4_ when burned. When wood products decompose in landfills, part of the carbon sequestrated is released as CH_4_. In these cases, models should estimate the global warming potential with the respective factors to estimate the climate change mitigation effect of wood products. This is taken into account when following the IPCC guidelines because landfill emissions of CO_2_ and non‐CO_2_ are reported by the wood product sector and the waste sector, respectively.

Model evolution shows a positive trend, with important characteristics like the *substitution effect* and *recycling* increasing their presence. When estimating present and future carbon stock, it is important to represent technological improvements and new wood uses, but time‐dependent parameters are also still unusual in wood product models (Table 4). Examples of models that provide time‐dependent parameters are the ones used in Harmon *et al*. (1996), Muller *et al*. (2004), Kurz *et al*. (2009) or Dymond (2012). The diversity of recent models is due to the increasing variety of purposes they are built for.

Notice that when applying models, some characteristics are intentionally avoided. The bucking allocation module is not necessary when using official statistical sources for input data since these sources already classify products. Recycling is also excluded when using official data sources because recycled products are included in the production categories. Disposal sites are intentionally avoided when nations report greenhouse gas emissions and removals to the UNFCCC. Additionally, the substitution effect is not estimated in UNFCCC reporting, because emissions from forest operations and industrial activity are reported by other sectors. However, these components may be essential when models are applied for other purposes. The bucking allocation module is very important when models are used to estimate the effect of management. Recycling should be included when wood product model inputs are produced by a forest growth model. The substitution effect is a key characteristic when estimating the mitigation effect of using wood products. The user needs to be aware of which model characteristics are relevant for a specific model application and select a model accordingly.

Recommendation for the use of specific models is difficult, but models classified in Group A merit recommendation due to their high scores both in their *structure* and *use* components. Models classified in Group C are only recommended to expert users due to their low scores in the *use* component. The models classified in Group C may be appropriate for some applications, but the absence of aids for their use may make it complicated to fully take advantage of them. Models in Group B may be appropriate for nonexpert users in applications like carbon reporting to the UNFCCC using the Tier 2 method. The selection of a model has to be done carefully according to the purposes of the study, since they may exclude important characteristics or include others which may be unnecessary. The Carbon Budget Modelling Framework for Harvested Wood Products (CBM‐FHWP) is an interesting model generator meant to develop new wood product models adapted to user needs. It was used to develop models like NFCMARS or BC‐HWPv1. The analysis and evaluation presented in this study could also be of interest for future model developers, since it summarizes model characteristics and compares available options for inclusion.

The main difficulty wood product models face is a lack of data. Relevant time‐ and location‐specific data regarding industrial processes is absent. Also, reliable data regarding the use phase, important to estimate product lifespan and removal rate, is generally lacking. Hence, wood product models heavily rely on assumptions. One consequence is that calibration and validation cannot be conducted. These conditions lead to uncertain estimates of the climate change mitigation effect of wood product use, and therefore weaken the climate mitigation claims of the forestry sector. Additional time‐ and location‐specific data on wood consumers' behaviour and other life cycle inventory data must become a priority to estimate the importance of wood products in a low‐carbon economy. A common effort from all stakeholders involved (e.g. forest owners, wood industries, recycling companies and related public entities) to improve monitoring and share data is due. Such efforts may strengthen the competitiveness of wood in comparison to other materials on the road to a low‐carbon economy.

## Supporting information


**Figure S1.** Effect of distribution functions for wood product removal on the carbon stock in wood products.
**Table S1.** Values describing the curves obtained using six different distribution functions to describe the removal of wood products from use.
**Data S1.** Effect of distribution functions for wood product removal on carbon stock.
