# Supplementary material for: Modelling carbon stocks and fluxes in the wood product sector: a comparative review
Source: Glob Chang Biol. 2016 Mar 4;22(7):2555–69. doi: 10.1111/gcb.13235 (PMC4949703; doi:10.1111/gcb.13235)
Supplement: Supplementary file 1 — Figure S1. Effect of distribution functions for wood product removal on the carbon stock in wood products. Table S1. Values describing the curves obtained using six different distribution functions to describe the removal of wood products from use. Data S1. Effect of distribution functions for wood product removal on carbon stock. [file GCB-22-2555-s001.docx]

# Supporting information

Supporting Information 1

Several distribution functions have been used in wood product models to simulate product removal from use. The selection of one or another affects carbon stock results. We compared the carbon stock in products for the six distribution functions encountered in the models: uniform, linear, exponential, logistic, normal and gamma. We simulated a constant annual product input of 1 ton carbon (tC) and a product half-life of 50 years, which means that after 50 years, 50% of this initial amount of product remains in use. In this exercise, we used the distributed approach and ran the model until a steady state was reached. Additional values needed to calculate the carbon retention curves, such as standard deviation in the case of normal distribution, have been extracted from literature when available; otherwise, they have been approximated.

Removal rates resulting from applying different distribution functions are compared in the left panel of Figure S1-1. Carbon stock estimations at steady state of products with 50 years of half-life produced at a constant rate of 1tC year^-1^ when applying different distributions are compared in the right panel of Figure S1-1. Values describing the curves obtained are compared in Table S1-1.

Table S1-1: Values describing the curves obtained using six different distribution functions to describe the removal of wood products from use. A fixed median (or half-life) of 50 years was chosen for this exercise to compare the effect of each distribution function.

| Distribution function | Median [50% of C left] (year) | 5% of C left (year) | Mean [average life] (year) | Mode [maximum rate of carbon loss] (year) |
| --- | --- | --- | --- | --- |
| Uniform | 50 | 50 | 50 | 51 |
| Linear | 50 | 95 | 50 | 0-100 |
| Weibull | 50 | 216 | 72 | 1 |
| Logistic | 50 | 121 | 55 | 42 |
| Normal | 50 | 74 | 50 | 50 |
| Gamma | 50 | 87 | 52 | 46 |


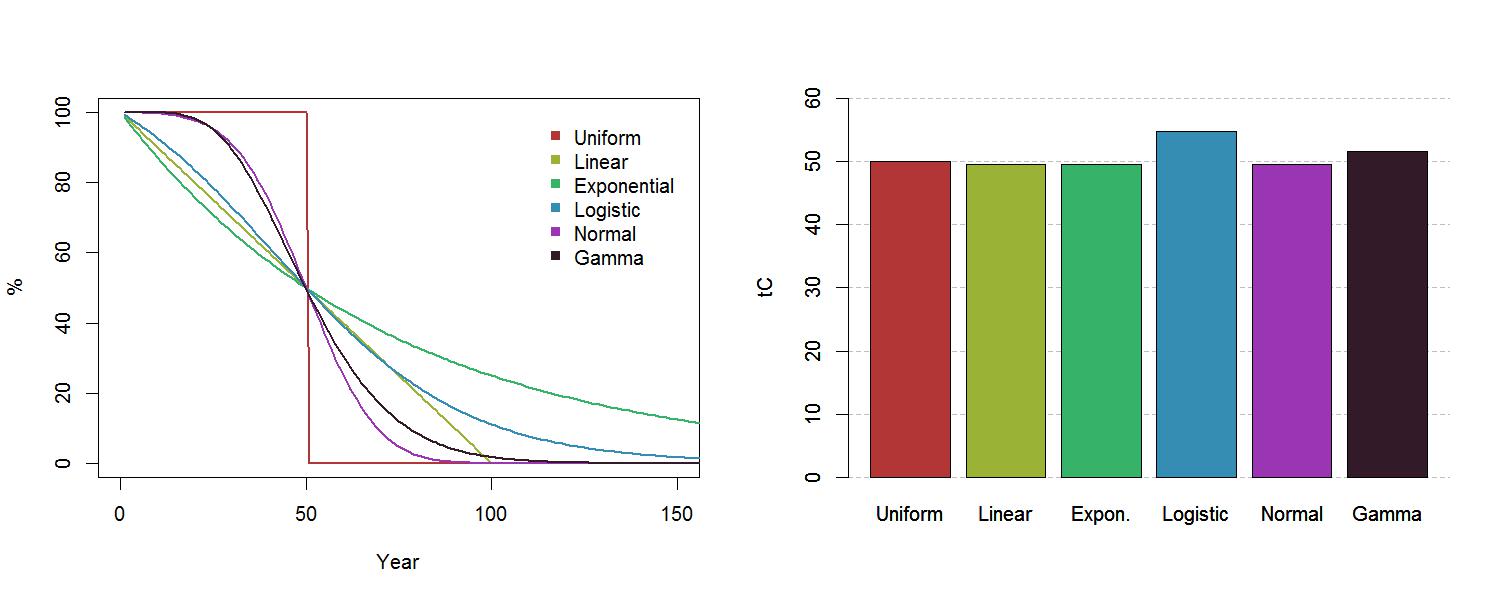


Figure S1-1: Effect of distribution functions for wood product removal on the carbon stock in wood products. The figure on the left compares the removal rate of wood products following different distribution functions. The figure on the right compares the carbon stock in wood products when using different distribution functions.
